# Celastrol Prevents Oxidative Stress Effects on FSHR, PAPP, and CYP19A1 Gene Expression in Cultured Human Granulosa-Lutein Cells

**DOI:** 10.3390/ijms22073596

**Published:** 2021-03-30

**Authors:** Rita Martín-Ramírez, Rebeca González-Fernández, Deborah Rotoli, Jairo Hernández, Pablo Martín-Vasallo, Angela Palumbo, Julio Ávila

**Affiliations:** 1 Laboratorio de Biología del Desarrollo, UD de Bioquímica y Biología Molecular and Centro de Investigaciones Biomédicas de Canarias (CIBICAN), Universidad de La Laguna, La Laguna, Av. Astrofísico Sánchez s/n, 38206 La Laguna, Tenerife, Spain; rmartira@ull.edu.es (R.M.-R.); refernan@ull.edu.es (R.G.-F.); deborah_rotoli@yahoo.it (D.R.); pmartin@ull.edu.es (P.M.-V.); 2 Institute of Endocrinology and Experimental Oncology (IEOS), CNR-National Research Council, 5-80131 Naples, Italy; 3 Centro de Asistencia a la Reproducción Humana de Canarias, 38202 La Laguna, Tenerife, Spain; jairoh@fivap.com (J.H.); apalumbo@fivap.com (A.P.)

**Keywords:** granulosa-lutein cells, oxidative stress, reactive oxygen species, reactive nitrogen species, celastrol

## Abstract

Regulation of oxidative stress (OS) is important to prevent damage to female reproductive physiology. While normal OS levels may have a regulatory role, high OS levels may negatively affect vital processes such as folliculogenesis or embryogenesis. The aim of this work was to study OS induced by glucose, a reactive oxygen species generator, or peroxynitrite, a reactive nitrogen species generator, in cultured human granulosa-lutein (hGL) cells from oocyte donors, analyzing expression of genes involved in oocyte maturation (FSHR, PAPP, and CYP19A1) and OS damage response (ALDH3A2). We also evaluated the effect of celastrol as an antioxidant. Our results showed that although both glucose and peroxynitrite produce OS increments in hGL cells, only peroxynitrite treatment increases ALDH3A2 and PAPP gene expression levels and decreases FSHR gene expression levels. Celastrol pre-treatment prevents this effect of peroxynitrite. Interestingly, when celastrol alone was added, we observed a reduction of the expression of all genes studied, which was independent of both OS inductors. In conclusion, regulation of OS imbalance by antioxidant substances such as celastrol may prevent negative effects of OS in female fertility. In addition to the antioxidant activity, celastrol may well have an independent role on regulation of gene expression in hGL cells.

## 1. Introduction

The role of oxidative stress (OS) in the regulation of human fertility has recently been recognized and associated with tissue injury and subsequent disruption of normal physiology in the female reproductive system, disturbing processes such as follicular development and maturation. While normal OS levels play an important regulatory role through different signaling pathways, high OS levels may negatively affect basic physiological processes including folliculogenesis, oocyte maturation, corpus luteum and uterine function, embryogenesis, embryo implantation, and fetoplacental development [1,2]. Under normal conditions, cells present a balance between oxidant and antioxidant agents. In some cases, this balance is disturbed by oxidative stress (OS) as a result of increased generation of reactive oxygen species (ROS), reactive nitrogen species (RNS), or a weaker antioxidant defense response [3,4]. OS can cause cellular injury involving proteins, lipids, and DNA, resulting in cellular damage and disease [3]. Factors such as lifestyle (alcohol, environmental exposures), obesity and aging, and fertility-related diseases like endometriosis and polycystic ovary syndrome, contribute to increased OS levels and enhance the impact of OS on female reproduction [4]. Previous studies in women undergoing fertility treatments showed a relationship between higher oxidative stress biomarker levels and lower oocyte fertilization potential [5]. Rosen et al. found a non-linear association between oxidative stress and reproductive outcomes in in vitro fertilization (IVF) patients, with higher rates of fertilization and pregnancy in women with middle levels of oxidative stress compared to women with lower or upper levels [6].

Cells have antioxidant mechanisms of defense to restore normal oxidative conditions. These mechanisms can be classified in multiple groups: enzymatic (i.e., superoxide dismutase SOD, peroxidase GPx, catalase CAT) and non-enzymatic (i.e., vitamin C, zinc, glutathione), primary (scavenger actions) or secondary defense (implicated in repair of biomolecules). As an example of a secondary defense response, lipid peroxidation and endoplasmic reticulum stress (ERS) that occur in an OS state promote transcription of target genes involved in OS resistance and cell survival [7,8,9]. One of these target genes is aldehyde dehydrogenase 3 member A2 (ALDH3A2), a nicotinamide adenine dinucleotide phosphate-dependent microsomal enzyme expressed in several tissues in response to ERS [10]. It is well documented that there is a link between ALDH3A2 expression and OS. ALDH3A2 KO mice show an increased OS response and upregulated transcription of OS-induced genes [11]. In chicken embryos, ALDH3A2 gene expression level is enhanced after addition of (−)-hydroxycitric acid and glucose [12]. In humans, ALDH3A2 expression is induced via upregulation of PGC-1α during fasting [13]. ALDH3A2 gene expression in human granulosa-lutein (hGL) cells correlates with age and infertility diagnosis, as a marker of OS status [14].

Follicle-stimulating hormone (FSH) is a glycoprotein secreted by the pituitary gland with a major role in promoting follicle development and in the regulation of ovarian function during folliculogenesis [15,16]. FSH is a major survival factor for antral follicles and has been suggested to improve granulosa cell (GC) resistance to oxidative stress during follicular atresia/apoptosis [17]. A novel role of FSH has been proposed in protecting ovarian GCs from oxidative injury via suppressing autophagy [18].

The FSH receptor (FSHR) is a seven-transmembrane domain protein that belongs to the δ group of the highly conserved subfamily of G protein-coupled receptors (GPCRs) [19,20]. FSHR is expressed in GCs [21]. The physiological responses to FSH are modulated by the activation of multiple target genes and microRNAs in GCs [22,23,24], such as luteinizing hormone (LH) receptor [25,26], autocrine factors [27], CYP19A1 [28], PAPP [29], and transcription factors [29].

However, few reports have evaluated the prosurvival effects of FSH on hGL cells undergoing OS [30]. Under OS conditions, FSH maintains mitochondrial integrity, restrains oxidative stress-induced mitophagy, prevents mitophagy-dependent hGL cell death, and protects hGL cells from mitophagic death by inhibiting the PINK1-Parkin pathway [30].

FSH binding to its receptor upregulates the expression of PAPP and CYP19A1 genes. PAPP-A and PAPP-A2 are metalloproteases that cleave insulin-like growth factor-binding protein 4 and 5 (IGFBP4 and IGFBP5), respectively [31,32]. PAPP-A is a metalloprotease associated with pregnancy and synthesized in many other circumstances in cells like osteoblasts, fibroblasts, and smooth muscle cells [33]. It has been demonstrated that GCs are a source of PAPP-A in the human ovary, suggesting that PAPP-A is a marker of ovarian follicle selection and corpus luteum formation [33]. In addition, an increment of PAPP-A gene expression was found in hGL cells after recombinant FSH treatment, reflecting IGFBP-4 proteolytic activity [34]. P450 aromatase (CYP19A1) catalyzes the conversion of androgens to estrogens [35,36], is located in the endoplasmic reticulum of estrogen-producing cells in the ovary, placenta, testis, brain, adipose tissue, liver, muscle, and hair follicles [37,38], and is regulated by FSH [39]. In addition, CYP19 is known as a novel mediator of T3- and FSH-induced follicular development [40]. It has been speculated that normal expression or overexpression of CYP19 mRNA could promote P450 aromatase activity, which would result in ovarian dysfunction [41].

In the context of oxidative stress regulation, it is of paramount importance to study antioxidant molecules that could play a role in the modulation of defense mechanisms and thus lead to the development of novel therapeutic drugs [42]. Celastrol, also known as tripterine, is a bioactive compound mainly isolated from *Tripterygium wilfordii*, with anti-tumoral [43,44,45], anti-inflammatory [46,47], antioxidant [45,48,49], anti-obesity [50], and anti-fibrotic [51] properties that has been approved for the treatment of asthma and neurodegenerative disease [52,53,54,55]. In skeletal muscle of diabetic rats, celastrol activates the AMPK-PGC1α-SIRT3 signaling pathway with resulting decrease of the OS status. Also, its antioxidative activity protects human retinal pigmented epithelial cells from H_2_O_2_-induced OS and apoptosis by activation of the SIRT3 signaling pathway [48,56].

In addition to its antioxidant role as a scavenger molecule, celastrol has other physiological effects on cells. Celastrol induces cell cycle arrest, apoptosis, and autophagy by the activation of the ROS/c-Jun N-terminal kinase (JNK) signaling pathway [57]. In colon cancer cells, celastrol has a cytotoxic effect on drug-resistance via ROS-dependent mechanisms [58]. Celastrol has a prooxidative activity causing DNA damage leading to cell cycle arrest and apoptosis induction [58]. A recent study showed that levels of malondialdehyde (MDA), superoxide anions, and Nox activity were enhanced and SOD activity was attenuated in collagen-induced arthritis (CIA) in rats, and these effects were reversed by celastrol treatment [59]. Despite the potential of celastrol as therapeutic drug [60], administration of such can cause side effects such as cardiotoxicity [61,62], hepatotoxicity [63], or infertility [64]. For this reason, analogues or less toxic chemical derivatives have been designed [65,66,67].

The purpose of this study was to investigate the effect of OS induced by glucose or peroxynitrite in cultured human granulosa-lutein cells by analyzing ALDH3A2, FSHR, PAPP, and CYP19A1 gene expression levels and to evaluate the protective effect of antioxidants such as celastrol.

## 2. Results

### 2.1. Effect of Glucose on Oxidative Stress

**Figure 1:**
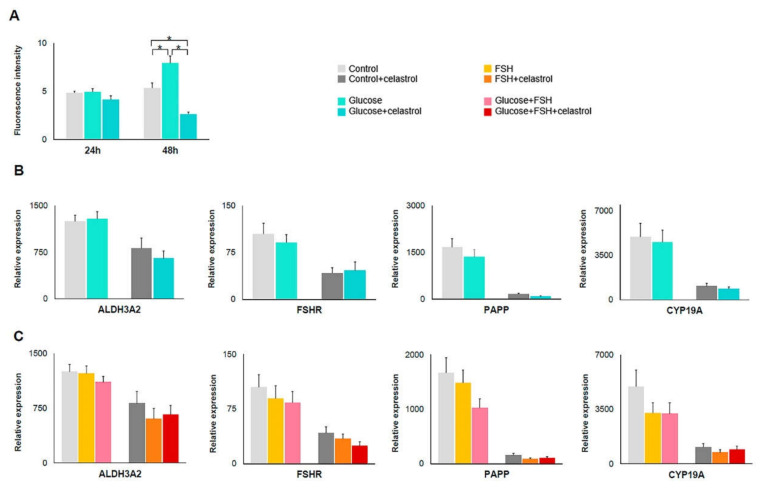
Oxidative stress (OS) levels and gene expression following glucose and/or celastrol treatments. (**A**) Histogram showing ROS levels in hGL cells after OS induction with glucose and with glucose plus celastrol; *n* = 3. (**B**) ALDH, FSHR, PAPP, and CYP19A1 gene expression levels in different cells treated with glucose (*n* = 27) and glucose plus celastrol (*n* = 18); (**C**) ALDH, FSHR, PAPP, and CYP19A1 gene expression levels in different cells treated with FSH, glucose plus FSH (*n* = 27), and its combination with celastrol (*n* = 18). Asterisks (*) indicate statistically significant differences.

ROS levels in hGL cells treated with glucose and glucose+celastrol were quantified by 2′,7′–dichlorofluorescin diacetate (DCFDA) assay at 24 and 48 h. Cells treated with glucose showed a 2% increase in OS levels at 24 h when compared to the control group. After 48 h of treatment, OS levels increased by 48% compared to controls (Figure 1A). The addition of celastrol reduced the glucose-induced increase in OS levels by 14% and 50% at 24 and 48 h, respectively.

Glucose-treated hGL cells showed no difference in the expression levels of ALDH3A2, FSHR, PAPP, and CYP19A1 genes compared to control cultures (Figure 1B; Table 1). The addition of FSH and/or celastrol to glucose-treated cells did not modify expression of the studied genes (Table 1; Figure 1B,C).

### 2.2. Effect of Peroxynitrite on Oxidative Stress

**Figure 2:**
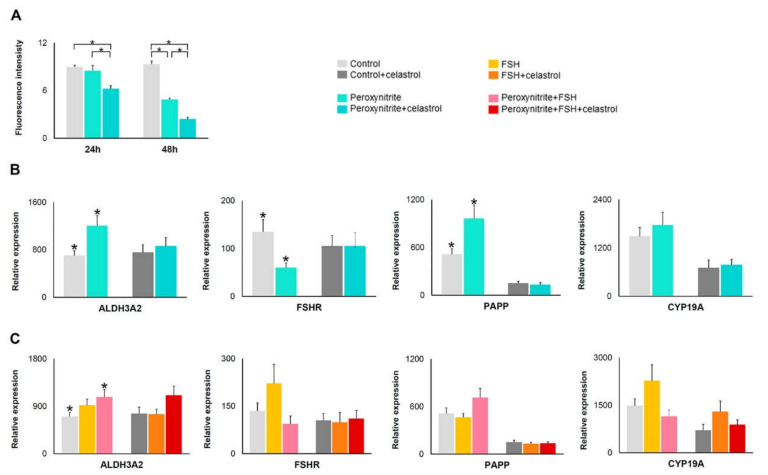
OS levels and gene expression in peroxynitrite and/or celastrol treatments. (**A**) Histogram showing OS levels in hGL cells after OS induction with peroxynitrite and with peroxynitrite plus celastrol; *n* = 3. (**B**) ALDH, FSHR, PAPP, and CYP19A1 gene expression levels in different cells treated with peroxynitrite (*n* = 26) and peroxynitrite plus celastrol (*n* = 18); (**C**) ALDH, FSHR, PAPP, and CYP19A1 gene expression levels in different cells treated with FSH, peroxynitrite plus FSH (*n* = 26), and its combination with celastrol (*n* = 18). Asterisks (*) indicate statistically significant differences compared to control.

In peroxynitrite-treated hGL cells, the DCFDA assay detects a decrease of total OS levels per well by 5% and 48% at 24 and 48 h, respectively, compared to control (Figure 2A). The addition of celastrol induced a further 30% and 74% decrease of OS generation at 24 and 48 h, respectively, compared to control (Figure 2A).

Gene expression analysis showed that ALDH3A2 and PAPP gene expression were higher, while expression of FSHR was lower in cells treated with peroxynitrite when compared to control cells (Table 2; Figure 2B). There was no change in CYP19A1 expression. The addition of celastrol blocked this effect of peroxynitrite on gene expression. (Table 2; Figure 2B). Expression levels of studied genes in FSH-treated hGL cells do not differ from control (Table 2; Figure 2C). The addition of FSH to peroxynitrite-treated cells induces a higher expression of ALDH3A2 (Table 2; Figure 2C), but not of the other two genes analyzed compared to control.

### 2.3. Effect of Celastrol

**Figure 3:**
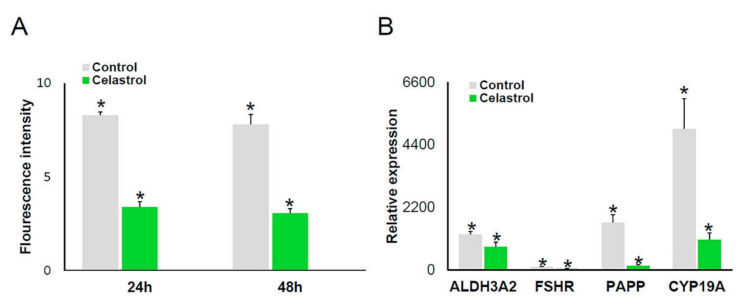
Effects of celastrol addition on OS and gene expression in cultured hGL cells. (**A**) Histogram showing OS levels in hGL cells after celastrol treatment; *n* = 3. (**B**) ALDH, FSHR, PAPP, and CYP19A1 gene expression in control (*n* = 27) and celastrol-treated cells (*n* = 18). Asterisks (*) indicate statistically significant differences compared to control.

The cellular OS detection assay showed a 60% decrease of OS levels in celastrol-treated cells compared to control (Figure 3A). In addition, Celastrol elicited a significant reduction in the expression level of all genes studied compared to control (Table 1; Figure 3B).

## 3. Discussion

Oxidative stress plays an important role in female infertility and has been involved in processes such as reduction of estradiol levels and follicle atresia via granulosa cell apoptosis [4,68,69], pathophysiology of pre-eclampsia [70], and implantation [70]. Thus, the possibility of modulating the OS status in ovarian follicles and reducing cellular damage generated by OS is a promising therapeutic approach to infertility and subfertility.

This study was designed to evaluate the effect of glucose, a metabolic accelerator, peroxynitrite, an oxidant substance, and celastrol, an antioxidant molecule, on oxidative stress in cultured human granulosa-lutein cells. Two methods were used to measure oxidative stress: (1) DCFDA assay; and (2) analysis of ALDH3A2, FSHR, PAPP, and CYP19A1 gene expression.

It is known that high glucose concentrations and consequent accelerated glucose metabolism generate OS by overproduction of ROS [71,72]. Our results showed a high OS level in glucose-treated cells as measured by the DCFDA assay, supporting the implication of glucose in OS generation in hGL cells. However, glucose treatment did not affect the expression levels of our selected reporter genes. A possible explanation is that under our experimental conditions, cells are able to respond to OS by other mechanisms [73]. Specifically, our experiments were performed using cells from healthy young women. It is possible that glucose treatment may have a different effect on cells from older women or women with pathologic conditions due to impaired cellular ability to respond to OS [14].

Peroxynitrite is a powerful oxidant (Table 2; Figure 2) that induces apoptosis and necrosis [74,75], lipid peroxidation and nitration [76,77], and DNA damage [78,79,80], and affects metabolic pathways such as glycolysis and oxidative phosphorylation [81,82]. Using the DCFDA assay, ROS levels were lower in peroxynitrite-treated hGL cells compared to control cultures, probably indicating a loss of viable cells induced by peroxynitrite. This result reflects the fact that the DCFDA assay measures the level of OS from live cells per well. On the other hand, this loss of viable cells did not affect gene expression quantitation because the 2^ΔCT^ method implies that results are expressed as a relative quantitation compared to actin. Our experiments showed that peroxynitrite treatment of hGL cells cause an increased expression of ALDH3A2 and PAPP and decreased expression of FSHR compared to control cultures, suggesting a role of peroxynitrite in triggering gene expression in response to cellular damage and affecting pathways involved in oocyte maturation. The addition of celastrol reverted the effect of peroxynitrite on gene expression, suggesting a role for celastrol as an antioxidant in hGL cells. Our results are in agreement with those of Zhou et al., who showed that granulosa cells from poor ovarian responders have elevated levels of peroxynitrite and decreased FSHR protein expression [83].

**Figure 4:**
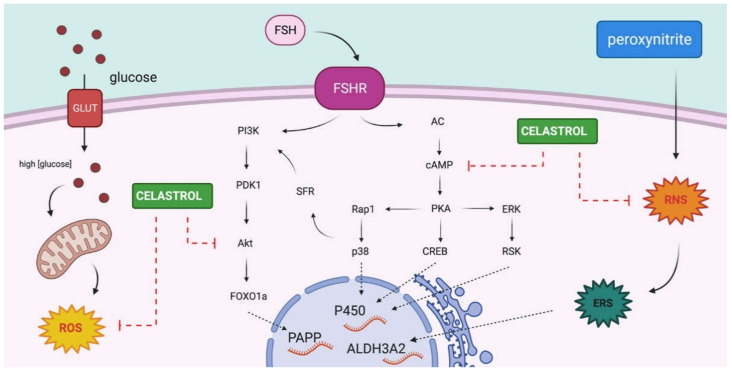
Scheme of OS inductors and celastrol actions on hGL cells. Proposed model for celastrol effects over PAPP and CYP19A1 gene expression and peroxynitrite effects over ALDH3A2 gene expression in hGL cells. Glucose addition produced higher ROS levels by an increase in the metabolism of glucose excess. Peroxynitrite treatment produced an increased RNS, causing endoplasmic reticulum stress (ERS). Celastrol acts as an antioxidant compound, reducing ROS and RNS levels. In addition, integrating our results with previously published data, we propose that celastrol may have a modulatory effect in pathways involved in PAPP and CYP19A1 gene expression (Created with BioRender.com; 15 March 2021).

The OS detection assay showed decreased ROS and RNS levels in celastrol-treated cells compared to control cultures after 24 and 48 h, suggesting an antioxidant role of celastrol under our experimental conditions. Interestingly, the effect of celastrol on the expression of our reporter genes suggests that celastrol may play an independent antioxidant regulatory role in hGL cells. In our experimental conditions, in celastrol-treated hGL cells, the expression levels of ALDH3A2, FSHR, PAPP, and CYP19A1 genes significantly decreased compared to control, an effect that seems to be independent of the antioxidant effect of celastrol. To our knowledge, this is the first evidence for a role of celastrol in the regulation of OS and gene expression in hGL cells. Despite the fact that celastrol contributions in the regulation of the signaling pathway are not well elucidated, it is known that celastrol plays a role in blocking the Akt/mTOR signaling pathway [84] and inhibition of cAMP accumulation [85]. PAPP and CYP19A1 gene expression are under FSHR regulation through different signaling pathways, such as PI3K/Akt or cAMP/PKA pathways, in which activation stimulates aromatase gene expression [41,86,87]. Figure 4 illustrates a proposed model for the mechanism of action of celastrol in hGL cells. This model integrates results shown in this article with current knowledge in signaling pathways activated by the FSH receptor along with the previously described action of celastrol on the activity of AKT and levels of cAMP. However, further studies are needed in order to confirm this hypothesis and to achieve a better understanding of this process.

## 4. Materials and Methods

### 4.1. Subjects

Human granulosa-lutein (hGL) cells were obtained from 64 healthy women between 18 and 27 years of age undergoing ovulation induction for the purpose of oocyte donation, classified as oocyte donors (OD), under a protocol approved by the Ethics Committee of the Universidad de La Laguna (CHUC_2018_694.2, 2018/21/01). Ovulation Induction Protocol.

Recombinant FSH (Gonal F, Serono, Madrid, Spain), combined with recombinant LH (Luveris, Serono, Madrid, Spain) or human menopausal gonadotropins (Menopur, Ferring, Madrid, Spain), were used for ovulation induction [88], adjusting doses to the individual patient’s response. Final oocyte maturation was triggered with 0.4 mg of leuprolide acetate (procrin solution, Abbvie, Spain) and 36 h later ultrasound-guided egg retrieval was performed.

### 4.2. Isolation of hGL Cells

Human granulosa-lutein cells were collected from follicular fluid obtained during ultrasound-guided transvaginal oocyte retrieval. After isolation of the oocytes from the follicular fluid, all the fluid from each woman was pooled and the hGL cells were lightly centrifuged. Cells were then washed in isolation medium (Medium 199, supplemented with sodium bicarbonate (3.7 g/L), penicillin (59 mg/L), streptomycin (100 mg/L), amphotericin B (25 mg/L), l-glutamine (0.29 g/L), and bovine serum albumin (0.1%)). A 50% Percoll gradient was used to separate GL cells from red blood cells. Leukocytes were removed using anti-CD45–coated magnetic beads (Dynabeads M-450 CD45; Dynal ASA, Oslo, Norway). Cellular viability was confirmed by trypan blue exclusion and was greater than 95% in all experiments.

### 4.3. Cell Culture and Treatments

Sterile 6-well dishes (Thermo Fisher Scientific, New York, NY, USA) were used for all cell cultures. Approximately 2.5 × 10^5^ viable cells were plated in each well. Cells were cultured for 48 h at 37 °C under 5% CO_2_ in McCoy’s 5A medium supplemented with l-glutamine (0.29 g/L), BSA (0.1%), penicillin (59 mg/L), streptomycin (100 mg/L), and amphotericin (25 mg/L).

#### 4.3.1. Analysis of OS Induced by Glucose in hGL Cells

Twenty-seven oocyte donors were included. The following conditions were set for each culture: control, +20 mM glucose, +20 mM glucose with the addition of 100 ng/mL FSH, +100 ng/mL FSH alone. FSH was added after 24 h of culture when indicated.

#### 4.3.2. Analysis of OS Cellular Damage Induced by Peroxynitrite in hGL Cells

Twenty-six oocyte donors were included. The following conditions were set for each culture: control, +0.1 mM peroxynitrite, +0.1 mM peroxynitrite with the addition of 100 ng/mL FSH, +100 ng/mL FSH. Peroxynitrite was added after 19 h of culture, and after 10 min incubation fresh medium was added. After the first 24 h of culture, FSH was added to cells when indicated. The peroxynitrite stock was prepared following the manufacturer’s instructions; final concentration was 16.43 mM diluted in NaOH 0.3 M.

#### 4.3.3. Analysis of the Protective Antioxidant Effect of Celastrol in hGL Cells

All experimental conditions were replicated with the addition of celastrol 1 μM. In 18 oocyte donors, the effect of celastrol alone (added at the beginning of the culture period) was tested.

### 4.4. Extraction of RNA

After 48 h culture, cells were washed with PBS and total RNA from each well was extracted using the Aurum total RNA mini kit (Bio-Rad Laboratories, Hercules, CA, USA) following the manufacturer’s instructions.

### 4.5. Synthesis of Complementary DNA

RNA was reverse transcribed using the iScript cDNA Synthesis kit (Bio-Rad Laboratories, Hercules, CA, USA) following the manufacturer’s instructions. Total RNA was reverse transcribed in 20 µL at 25 °C for 5 min and 42 °C for 30 min. Reaction was stopped by heating at 85 °C for 5 min.

### 4.6. Quantitative Reverse Transcription-Polymerase Chain Reaction (qRT-PCR)

Relative expression levels of studied genes were measured by qRT-PCR using the following primers: ALDH3A2 (GCCTATATTCAGCCACAGC, ATAATCACAGCATTTCCTGC), FHSR (ACCAAGCTTCGAGTCATCC, CATCTGCCTCTATCACCTCC), CYP19A1 (AGAAAAAAGACGCAGGATTTC, CTCTTGTCAGGTCACCACG), and PAPP (GGGTAGAGAGAGTTGTCTGCAC, TAATTGTCTCCCATGAAGGG) relative to the housekeeping β-actin gene (CTTCCTTCCTGGGCATGG, GCCGCCAGACAGCACTGT). PCRs were carried out in a Bio-Rad CFX96 real-time PCR system (Bio-Rad Laboratories, Hercules, California). The amplification reactions were performed in a 10 µL final volume containing 2× Sso Fast Eva Green Supermix (100 mmol/L KCl, 40 mmol/L Tris-HCl pH 8.4, 0.4 mmol/L of each nucleoside triphosphate, iTaq DNA polymerase 50 U/mL, 6 mmol/L MgCl_2_, SYBR Green I, 20 nmol/L fluorescein, and stabilizers (Bio-Rad Laboratories)) and 0.4 µmol/L of each primer.

Multiple water blanks were included, and each sample was analyzed in triplicate. The thermal profile used was as follows: after 30 s of initial denaturation at 95 °C, 45 cycles of PCR were performed at 95 °C for 5 s and 59 °C for 5 s. Finally, a melting curve program at 65 °C to 95 °C was carried out with a heating rate of 0.1 °C/s and read every 0.5 °C. Expression levels of the genes studied are presented as individual data points as 2^ΔCT^ [89].

### 4.7. Oxidative Stress Assay

The cellular OS level was evaluated using 2′,7′–dichlorofluorescin diacetate (DCFDA, also known as H2DCFDA)–Cellular Reactive Oxygen Species Detection Assay Kit (Abcam, Cambridge, England). This kit allows measurement of DFC fluorescence, a subproduct of DCFDA oxidation produced by ROS or RNS [58]. Cells were seeded out in clear-bottomed black-sided 96-well plates with a density of 25 × 10^3^ cells per well. The following culture conditions were used: control, +20 mM glucose, +0.1 mM peroxynitrite with or without the addition of 1 μM celastrol. After 24 and 48 h of culture, OS level was measured using 45 μM DCFDA following the manufacturer’s instructions. Briefly: cells were stained with DCFDA at 37 °C for 45 min and fluorescence was measured (Ex/Em = 485/535 nm) in a microplate reader (DTX 800, Beckman Coulter) after removal of DCFDA and addition of washing buffer.

### 4.8. Statistical Analysis

Statistical analysis was performed with the SPSS 23 software (IBM Corporation, Somers, NY, USA) using Student’s *t*-test to carry out comparisons between treatments. Descriptive statistics (mean and standard error (SE)) are reported. A *p*-value of <0.05 was considered statistically significant.

## 5. Conclusions

This work shows that both peroxynitrite (a direct OS inductor) and glucose (an indirect OS metabolic inductor which mimics a high carbohydrate diet) produce significant increase of OS levels in hGL cells. Peroxynitrite-induced alterations on gene expression levels were reverted by celastrol addition and further experiments are warranted to further assess the protective effects of celastrol and its underlying mechanisms.

## Figures and Tables

**Table 1:** Effect of glucose, FSH, and/or celastrol treatment on studied gene expression levels. Gene expression levels in hGL cells treated with glucose and its combination with FSH and/or celastrol. Results were determined by qRT-PCR and are expressed as mean ± standard error; *n* = 27. Gene expression values are ×10^5^ relative to β-actin expression.

	Genes			
Group	*ALDH3A2*	*FSHR*	*PAPP*	*CYP19A1*
**Control**	1250 ± 103	105 ± 17	1668 ± 279	4970 ± 1062
**Glucose**	1290 ± 118	91 ± 13	1359 ± 236	4556 ± 947
**FSH**	1226 ± 103	89 ± 17	1486 ± 236	3259 ± 666
**Glu + FSH**	1108 ± 79	84 ± 15	1022 ± 171	3217 ± 714
**Control + Celast**	816 ± 164	42 ± 9	156 ± 35	1067 ± 239
**Glucose + Celast**	650 ± 123	46 ± 14	92 ± 20	864 ± 154
**FSH + Celast**	604 ± 145	34 ± 6	87 ±2 0	731 ± 184
**Glu + FSH + Celast**	661 ± 129	24 ± 6	108 ± 23	924 ± 223

**Table 2:** Effect of peroxynitrite, FSH, and/or celastrol treatment on studied gene expression levels. Gene expression levels in hGL cells treated with peroxynitrite and its combination with FSH and/or celastrol. Results were determined by qRT-PCR and are expressed as mean ± standard error; *n* = 26. Gene expression values are ×10^5^ relative to β-actin expression. Asterisks (*) and daggers (†) indicate statistically significant differences compared to control.

	Genes			
Group	*ALDH3A2*	*FSHR*	*PAPP*	*CYP19A1*
**Control**	704 ± 29 *^,†^	135 ± 26 *	514 ± 69 *	1486 ± 221
**Peroxynitrite**	1204 ± 173 *	60 ± 10 *	961 ± 170 *	1771 ± 312
**FSH**	918 ± 120	221 ± 61	465 ± 48	2273 ± 508
**Perox + FSH**	1074 ± 144 ^†^	93 ± 26	713 ± 117	1150 ± 206
**Control + Celast**	757 ± 127	105 ± 22	151 ± 25	707 ± 193
**Perox + Celast**	861 ± 144	104 ± 28	133 ± 26	778 ± 138
**FSH + Celast**	746 ± 98	99 ± 31	130 ± 20	1299 ± 336
**Perox + FSH + Celast**	1106 ± 177	110 ± 26	136 ± 18	889 ± 151

## Data Availability

All data presented in this article can be obtained from Authors, upon request.

## Abbreviations

ALDH3A2	Aldehyde dehydrogenase 3 member A2
CYP19A1	P450 aromatase for estrogen production
DCFDA	2′,7′–dichlorofluorescin diacetate
ERS	Endoplasmic reticulum stress
FSH	Follicle stimulating hormone
FSHR	Follicle stimulating hormone receptor
GC	Granulosa cells
hGL	Human granulosa-lutein cells
OD	Oocyte donors
OS	Oxidative stress
PAPP	Pregnancy associated plasma protein
RNS	Reactive nitrogen species
ROS	Reactive oxygen species
